# Physical activity attenuates postprandial hyperglycaemia in homozygous *TBC1D4* loss-of-function mutation carriers

**DOI:** 10.1007/s00125-021-05461-z

**Published:** 2021-04-29

**Authors:** Theresia M. Schnurr, Emil Jørsboe, Alexandra Chadt, Inger K. Dahl-Petersen, Jonas M. Kristensen, Jørgen F. P. Wojtaszewski, Christian Springer, Peter Bjerregaard, Søren Brage, Oluf Pedersen, Ida Moltke, Niels Grarup, Hadi Al-Hasani, Anders Albrechtsen, Marit E. Jørgensen, Torben Hansen

**Affiliations:** 1 Novo Nordisk Foundation Center for Basic Metabolic Research, Faculty of Health and Medical Sciences, University of Copenhagen, Copenhagen, Denmark; 2 The Bioinformatics Centre, Department of Biology, University of Copenhagen, Copenhagen, Denmark; 3 Institute for Clinical Biochemistry and Pathobiochemistry, German Diabetes Center (DDZ), Leibniz Center for Diabetes research at the Heinrich-Heine-University Duesseldorf, Medical Faculty, Duesseldorf, Germany; 4 German Center for Diabetes Research (DZD), Duesseldorf, Germany; 5 National Institute of Public Health, University of Southern Denmark, Odense, Denmark; 6 Steno Diabetes Center Copenhagen, Gentofte, Denmark; 7 Section of Molecular Physiology, Department of Nutrition, Exercise and Sports, Faculty of Science, University of Copenhagen, Copenhagen, Denmark; 8 Medical Research Council Epidemiology Unit, University of Cambridge, Cambridge, UK; 9 Greenland Center for Health Research, University of Greenland, Nuuk, Greenland

**Keywords:** Arctic, Gene-environment interaction, Lifestyle therapy, Physical activity, Postprandial hyperglycaemia, *TBC1D4* loss-of-function

## Abstract

**Aims/hypothesis:**

The common muscle-specific *TBC1D4* p.Arg684Ter loss-of-function variant defines a subtype of non-autoimmune diabetes in Arctic populations. Homozygous carriers are characterised by elevated postprandial glucose and insulin levels. Because 3.8% of the Greenlandic population are homozygous carriers, it is important to explore possibilities for precision medicine. We aimed to investigate whether physical activity attenuates the effect of this variant on 2 h plasma glucose levels after an oral glucose load.

**Methods:**

In a Greenlandic population cohort (*n* = 2655), 2 h plasma glucose levels were obtained after an OGTT, physical activity was estimated as physical activity energy expenditure and *TBC1D4* genotype was determined. We performed *TBC1D4*–physical activity interaction analysis, applying a linear mixed model to correct for genetic admixture and relatedness.

**Results:**

Physical activity was inversely associated with 2 h plasma glucose levels (β[main effect of physical activity] −0.0033 [mmol/l] / [kJ kg^−1^ day^−1^], *p* = 6.5 × 10^−5^), and significantly more so among homozygous carriers of the *TBC1D4* risk variant compared with heterozygous carriers and non-carriers (β[interaction] −0.015 [mmol/l] / [kJ kg^−1^ day^−1^], *p* = 0.0085). The estimated effect size suggests that 1 h of vigorous physical activity per day (compared with resting) reduces 2 h plasma glucose levels by an additional ~0.7 mmol/l in homozygous carriers of the risk variant.

**Conclusions/interpretation:**

Physical activity improves glucose homeostasis particularly in homozygous *TBC1D4* risk variant carriers via a skeletal muscle TBC1 domain family member 4-independent pathway. This provides a rationale to implement physical activity as lifestyle precision medicine in Arctic populations.

**Data repository:**

The Greenlandic Cardio-Metabochip data for the Inuit Health in Transition study has been deposited at the European Genome-phenome Archive (https://www.ebi.ac.uk/ega/dacs/EGAC00001000736) under accession EGAD00010001428.

**Graphical abstract:**

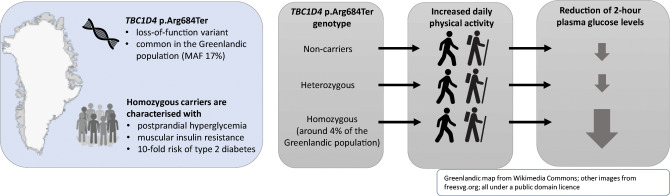

**Supplementary Information:**

The online version contains peer-reviewed but unedited supplementary material available at 10.1007/s00125-021-05461-z.

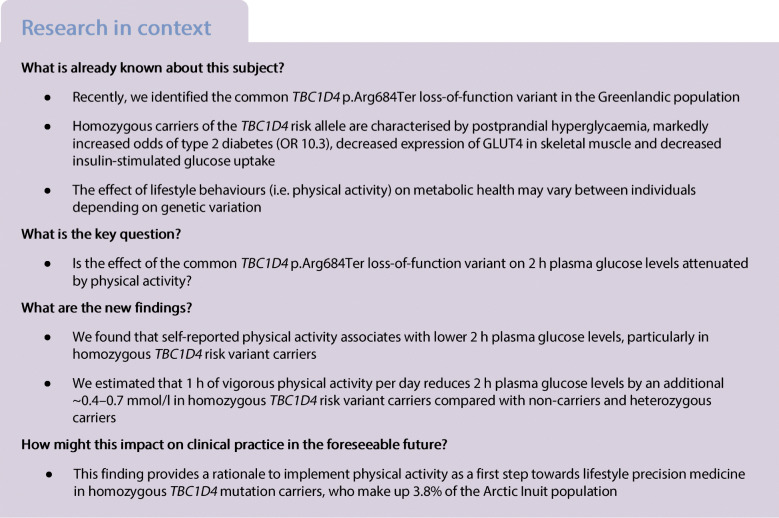



## Introduction

Type 2 diabetes is a growing global health problem and its prevalence is also rapidly increasing in Greenland and in other Arctic populations [1–3]. Recently, a common *TBC1D4* p.Arg684Ter loss-of-function variant that defines a specific subtype of non-autoimmune diabetes characterised by elevated postprandial glucose levels has been described. The variant was identified initially in the Greenlandic population (minor allele frequency of 17%, and 3.8% are homozygous carriers) and later described in the North American Inuit population with similar allele frequency [4, 5]. Homozygous carriers are characterised by postprandial hyperglycaemia (on average these individuals had 3.8 mmol/l higher 2 h plasma glucose during an OGTT), impaired glucose tolerance and 10.3-fold increased odds of developing type 2 diabetes [4]. The mutation is estimated to account for more than 10% of all cases of type 2 diabetes in Greenland and other Artic Inuit populations [4, 5]. Based on the variant’s high frequency and the clinical characterisation of homozygous *TBC1D4* risk variant carriers, it has been suggested that diagnostic strategies for type 2 diabetes should include 2 h OGTT and/or *TBC1D4* genotype risk stratification in Arctic populations [5–7].

There is a plausible pathophysiological explanation for the observed clinical characteristics of homozygous *TBC1D4* risk variant carriers. *TBC1D4* encodes a Rab-GTPase-activating protein (RabGAP) that represents a key factor in the insulin-stimulated GLUT4 translocation process [8]. Previously, we observed decreased GLUT4 content in skeletal muscle of homozygous carriers and speculate that this likely contributes to postprandial hyperglycaemia via a possible lowered glucose uptake in skeletal muscle [4]. Experiments in mice have shown that knocking down *Tbc1d4* causes a decrease in basal plasma glucose and a decrease in insulin-stimulated glucose uptake in muscle and adipose tissue [9]. Regular exercise training has been shown to regulate the phosphorylation of TBC1 domain family member 4 (TBC1D4) in skeletal muscle from individuals with type 2 diabetes, indicating a role for RabGAP in the insulin-sensitising effects of exercise training [10]. Importantly, regular physical activity is widely accepted as a useful means for both the prevention and treatment of metabolic diseases [11, 12] but the underlying mechanisms of the beneficial impact of physical activity on metabolic health are not yet fully understood.

It is not currently known whether daily physical activity especially benefits indigenous Arctic individuals with a muscular *TBC1D4* deficiency. Such knowledge might provide a powerful treatment option to effectively lower postprandial glucose levels and prevent type 2 diabetes in these populations and might provide insights into molecular mechanisms. Motivated by this, our main aim was to estimate the effect of daily physical activity on the association between the common *TBC1D4* p.Arg684Ter loss-of-function variant and postprandial plasma glucose levels during a 2 h OGTT in Greenlandic individuals. As secondary outcomes, we also examined whether daily physical activity reduces the odds of developing type 2 diabetes and has beneficial effects on other cardiometabolic traits in homozygous carriers of this loss-of-function variant. To our knowledge, this is the first study investigating the effect of gene–lifestyle interactions on cardiometabolic traits in Artic populations.

## Methods

### Study population

The Greenlandic cohort (*n* = 3115), also known as the Inuit Health in Transition study, is a population-based study of around 9% of the adult Greenlandic population. Data was collected after inviting a random sample of adults aged ≥18 years to participate in the population health survey in Greenland in 2005–2010. Pregnant women were not enrolled. Details about the health survey and data collection method are described in detail elsewhere [2]. Prior to participation, informed consent was obtained from all individuals included into the study. The study was approved by the Commission for Scientific Research in Greenland (project 2011-13, ref. no. 2011-056978, and project 2013-13, ref.no. 2013-090702) and was conducted in accordance with the principles of the Declaration of Helsinki.

### Cardiometabolic traits and definition of type 2 diabetes

All participants without known diabetes (at time of the examination) underwent a standardised 75 g, 2 h OGTT after an overnight fast of at least 8 h. Plasma glucose was analysed with the Hitachi 912 system (Roche Diagnostics). Measurements of secondary tested cardiometabolic traits are described in the electronic supplementary material (ESM) Methods. Type 2 diabetes was classified according to WHO criteria [13].

### Physical activity assessment

Information on daily physical activity was collected using an interviewer-administrated version of the International Physical Activity Questionnaire (long version) that was modified and adapted to Arctic living conditions (*n* = 3004 individuals of the entire cohort) as described in ESM Methods. Physical activity energy expenditure was calculated [14]. This questionnaire was found to be a valid measure for overall physical activity energy expenditure among adult Inuit in Greenland [14]. We based our primary main analysis on questionnaire-derived physical activity for enhanced power of our statistical analysis since this allowed us to include as many individuals as possible with valid information on physical activity, *TBC1D4* p.Arg684Ter genotype and 2 h plasma glucose concentrations (*n* = 2655). We performed sensitivity analysis in a subset of individuals (*n* = 1388) where physical activity energy expenditure was also assessed objectively by combined heart rate and acceleration monitors (ActiHeart; CamNTech, Cambridge, UK) to evaluate whether magnitude and direction of interaction effects were comparable between subjectively and objectively assessed physical activity. We included individuals with a monitor wear time of at least 48 h (*n* = 1544 individuals in the entire cohort) for this sensitivity analysis. Due to study logistics, limited time at each study location and a finite stock of monitors, not all participants were given an ActiHeart monitor and the length of recordings from some participants was shorter [14, 15]. A full description of the population-specific calibration of the heart rate–energy equation and assessment of physical activity energy expenditure is documented elsewhere [14–16]. We translated the effect sizes derived from the interaction models (β[interaction]) into aspects of intensity and type of activity to ensure relevance in the Greenlandic cohort. For this, we derived metabolic equivalent task (MET) activities based on the definition by Ainsworth et al [17] and the assumption that an increase in physical activity energy expenditure of 10 kJ kg^−1^ day^−1^ is equivalent to about 1 h intensity of 3.5 MET per day (e.g. moderate-paced walking at 2.8–3.2 miles per hour when walking for transportation on a firm surface) compared with spending this hour at rest. We also derived the equivalent to a high-intensity activity of 9.5 MET per day commonly performed by Greenlanders, namely hiking with hunting gear, compared with spending this hour at rest [17].

### Genotyping

Participants from the Greenlandic cohort (*n* = 3115) were genotyped by the Cardio-Metabochip, a custom iSelect genotyping array of 196,725 SNPs for genetic studies of metabolic, cardiovascular and anthropometric traits [18], on the Illumina HiScan platform (Illumina, San Diego, CA, USA). Genotypes were called using the GenCall module (version 1.9.4) of GenomeStudio software (version 2011.1; Illumina). During quality control using PLINK software tools (version 1.9; https://www.cog-genomics.org/plink/) [19] we removed a few individuals with mislabelled sex and high rate of missing data (>2% of all SNPs with a minor allele frequency >0.01), leaving 3074 individuals for which array genotyping was successful accomplished. The p.Arg684Ter nonsense polymorphism in *TBC1D4*, rs61736969, was genotyped in all participants (*n* = 3040) by applying the KASP genotyping assay (LGC Genomics, UK).

### Statistical analysis

Overall, 2655 individuals had complete information on questionnaire-derived physical activity, *TBC1D4* p.Arg684Ter genotype and 2 h plasma glucose levels, and hence were included in our primary analysis (ESM Fig. 1). We tested for a gene–environment interaction effect between *TBC1D4* p.Arg684Ter genotype and physical activity on 2 h plasma glucose using a linear mixed model implemented in GMMAT (version 1.0.3) [20]. We used a linear mixed model to correct for admixture and relatedness (see Eq. 1 in ESM Methods). We applied a gene–environment model with 2 h plasma glucose as response, with main effect terms for physical activity and *TBC1D4* p.Arg684Ter genotype, and an additional effect term for interaction between physical activity and *TBC1D4* p.Arg684Ter. Our group has shown previously that the effect of the *TBC1D4* p.Arg684Ter risk variant on 2 h plasma glucose levels is almost entirely recessive but there is some effect of being heterozygous [4]. In the primary analytical model, we therefore modelled the *TBC1D4* p.Arg684Ter effects as recessive. Including the interaction effect term in the model allowed us to test if the effect of physical activity is different for homozygous *TBC1D4* p.Arg684Ter risk variant carriers compared with heterozygous carriers and non-carriers and allowed us to estimate the size of this difference (β[interaction]).

Quantitative traits were transformed using sex-specific rank-based inverse normal transformations to ensure that they follow a normal distribution as assumed in the applied model. SEs, effect sizes (in SD) and *p* values are reported from transformed analyses. We also report effect sizes from analyses of untransformed traits. We applied the Wald test implemented in GMMAT [20] for our primary analysis (see Eq. 1 in ESM Methods), assuming a significance level of 0.05. We also explored interaction analyses of *TBC1D4* with physical activity on additional cardiometabolic traits, assuming a significance level of 0.05 (applying Eq. 1, ESM Methods). Furthermore, we tested for a *TBC1D4*–physical activity interaction on type 2 diabetes using a logistic model implemented in GMMAT (ESM Methods) [20]. We excluded individuals with known diabetes at the date of examination from all analyses and individuals taking lipid-lowering medications were removed in analysis of lipid measures.

Finally, to compare values of 2 h plasma glucose levels for different physical activity levels for non-carriers, heterozygous carriers and homozygous carriers of the *TBC1D4* p.Arg684Ter risk variant, we predicted values for each of the three possible genotypes for a range of physical activity levels. For this, we used the primary analytical model with the effect sizes estimated based on untransformed 2 h plasma glucose levels. To obtain predictions relative to an average non-carrier individual with a physical activity level of 50 kJ kg^−1^ day^−1^, we subtracted 50 kJ kg^−1^ day^−1^ multiplied by β_1_ (β[main effect for physical activity]) from all predicted values.

## Results

**Fig. 1:**
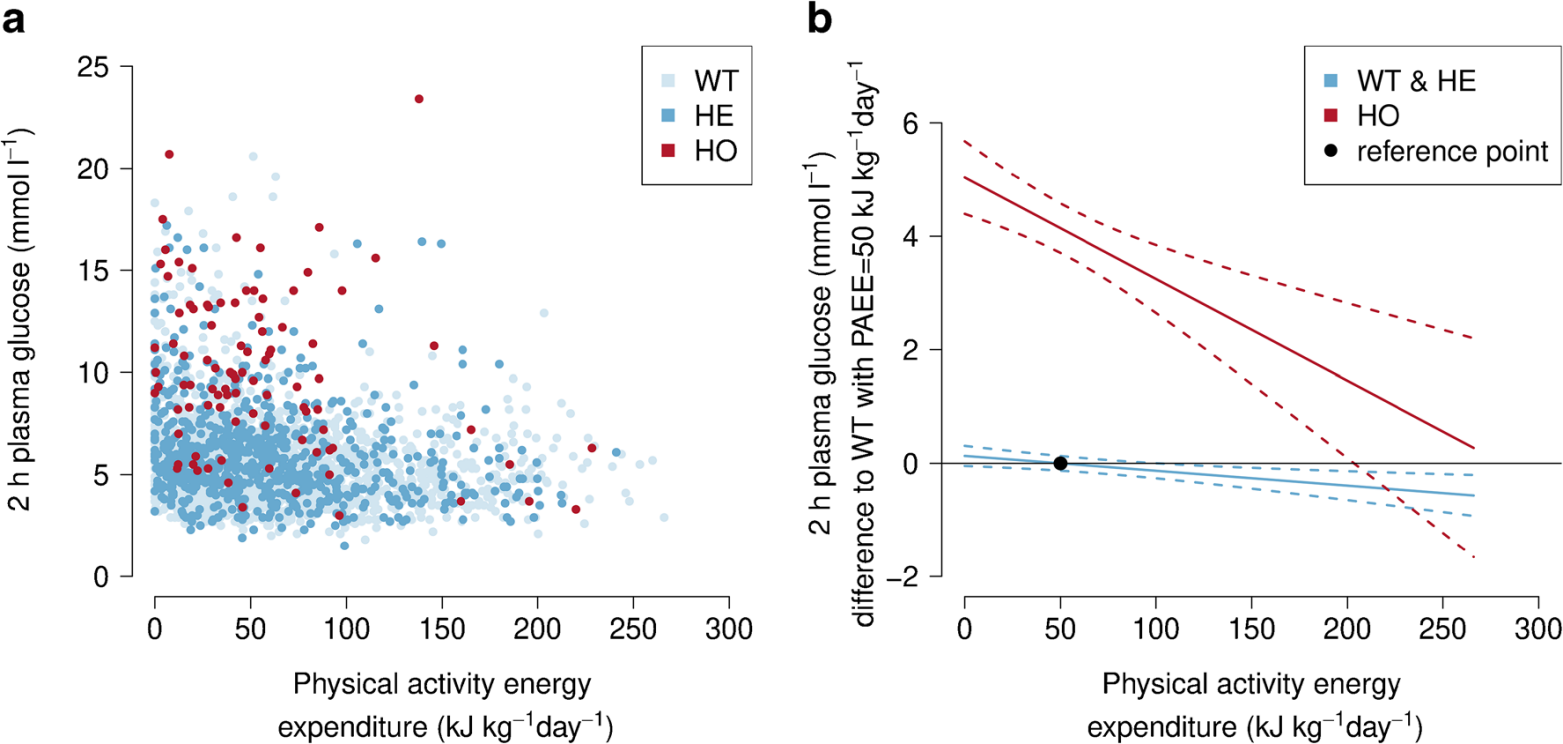
Physical activity attenuates the effect of the common muscle-specific *TBC1D4* p.Arg684Ter loss-of-function variant on 2 h plasma glucose levels in 2655 Greenlanders. (**a**) Raw data points (physical activity measured as physical activity energy expenditure on the *x*-axis and 2 h plasma glucose levels on the *y*-axis) stratified by *TBC1D4* p.Arg684Ter genotype. (**b**) Illustration of the *TBC1D4*–physical activity interaction effect on 2 h plasma glucose levels relative to a standard individual in Greenlanders. For each individual, the respective mean physical activity energy expenditure per day is plotted on the *x*-axis. The *y*-axis displays the difference in 2 h plasma glucose levels for each individual compared with a standard individual (defined as non-carrier having an assumed physical activity energy expenditure of 50 kJ kg^−1^ day^−1^ [~ median of the cohort, illustrated by the black circle]). The predictions were performed using the estimated effect sizes from the primary analytical model and 95% CIs are shown. HE, heterozygous *TBC1D4* p.Arg684Ter variant carriers; HO, homozygous *TBC1D4* p.Arg684Ter variant carriers; PAEE, physical activity energy expenditure; WT, non-carriers

Clinical characteristics of the Greenlandic individuals included in the presented analyses are shown in ESM Table 1. We found that homozygous *TBC1D4* p.Arg684Ter variant carriers had highly elevated levels of 2 h plasma glucose compared with non-carriers (β[main effect of being homozygous *TBC1D4* risk variant carrier] = 4.2 mmol/l, *p* = 1.2 × 10^−40^, *n* = 2655), which is consistent with our previous observation [4]. Furthermore, physical activity was inversely associated with 2 h plasma glucose levels (β[main effect of physical activity] = −0.0033 [mmol/l] / [kJ kg^−1^ day^−1^], *p* = 6.5 × 10^−5^, *n* = 2655, Fig. 1a).

The effect of physical activity on 2 h plasma glucose levels was significantly greater among homozygous carriers of the *TBC1D4* variant compared with heterozygous carriers and non-carriers (β[interaction] = −0.015 [mmol/l] / [kJ kg^−1^ day^−1^], *p* = 0.0085, *n* = 2655; Fig. 1b, Table 1 and ESM Fig. 2a). Additional adjustment for BMI did not change the magnitude of interaction (β[interaction] =   −0.015 [mmol/l] / [kJ kg^−1^ day^−1^], *p* = 0.011, *n* = 2635). Importantly, we confirmed the magnitude and direction of the interaction effect in the subset of individuals with available information on objectively assessed physical activity (β[interaction] = −0.024 [mmol/l] / [kJ kg^−1^ day^−1^], *p* = 0.45, *n* = 1388; Table 1). We did not find any significant difference in physical activity energy expenditure when comparing homozygous *TBC1D4* p.Arg684Ter carriers, heterozygous carriers and non-carriers in the Greenlandic population (*p* = 0.65). Additional adjustment of the primary analytical model for total energy consumption did not change the magnitude of β(interaction) in individuals with available information on total energy consumption (data not shown).

**Table 1:** Effect of the *TBC1D4* p.Arg684Ter–physical activity interaction on 2 h plasma glucose levels in Greenlandic Inuit

Physical activity energy expenditure assessment	Genetic model	Tested parameter^a^	Effect (SD)^b^	SE^b^	*p* value^b^	Effect (β[interaction], [mmol/l] / [kJ kg^−1^ day^−1^])^c^	Estimated effect (mmol/l) for ~1 h of moderate-paced walking instead of spending this hour at rest (3.5 MET activity^d^ per day)	Estimated effect (mmol/l) for ~1 h of hiking with hunting gear instead of spending this hour at rest (9.5 MET activity^d^ per day)
International Physical Activity Questionnaire (*n* = 2655)	Recessive (primary)	*β*_*HOxa*_ (β[(interaction])	−0.0051	0.00190	0.0085	−0.0150	−0.2	−0.4
	Additive	*β*_*gxa*_	−0.0021	0.00067	0.0014	−0.0056	−0.1	−0.2
Combined heart rate and acceleration (*n* = 1388)	Recessive (primary)	*β*_*Hoxa*_ (β[interaction])	−0.0042	0.00560	0.4500	−0.0240	−0.2	−0.7
	Additive	*β*_*gxa*_	−0.0040	0.00180	0.0270	−0.0140	−0.1	−0.4

^a^See Eq. 1 in ESM Methods

^b^Effect (in SD of the study population), SE and *p* value are from the analysis of transformed traits

^c^Effect in (mmol/l) /(kJ kg^−1^ day^−1^) is from analysis of untransformed traits

^d^MET activities were previously defined by Ainsworth et al [17] and were derived based on the assumption that a 10 kJ kg^−1^ day^−1^ increase in physical activity energy expenditure is equivalent to about 1 h intensity of 3.5 MET per day (e.g. moderate-paced walking at 2.8–3.2 miles per hour, when walking for transportation on a firm surface) compared with spending this hour at rest. We also derived a high-intensity activity of 9.5 MET per day, namely 1 h of hiking with hunting gear, compared with spending this hour at rest

To be clinically useful, it is important to translate the effect sizes derived from the interaction models (β[interaction]) into real-life settings. Our results suggest that for every 10 kJ kg^−1^ day^−1^ increase in physical activity energy expenditure, concentrations of 2 h plasma glucose were reduced by an additional ~0.2 mmol/l in homozygous *TBC1D4* p.Arg684Ter carriers compared with heterozygous carriers and non-carriers (Table 1). This difference in physical activity corresponds to about 1 h of activity at an intensity of 3.5 MET per day (e.g. moderate-paced walking) compared with spending this hour at rest [17]). More extremely, 1 h daily of a high-intensity activity commonly performed by Greenlanders (e.g. hiking with hunting gear), compared with spending this hour at rest [17], decreased 2 h plasma glucose levels by an additional ~0.4–0.7 mmol/l in homozygous *TBC1D4* p.Arg684Ter carriers compared with heterozygous carriers and non-carriers (based on self-reported and objective assessed physical activity, respectively; Table 1).

We also investigated the effect of the *TBC1D4* p.Arg684Ter–physical activity interaction on 2 h plasma glucose levels under a standard additive interaction model (ESM Methods) and found similar or stronger interaction effects compared with the applied primary recessive interaction model. Remarkably, the additive model showed a significant interaction effect in the sensitivity analysis of the subset including individuals with objectively measured physical activity (*p* = 0.027, Table 1). To ensure that the results of our primary analysis were not confounded by admixture, we stratified individuals included into the analysis by different degrees of European admixture (0–5%, 5–20%, 20–35% and >35% European admixture) and performed interaction analysis by applying the primary model to each subgroup separately. None of the interaction effects for the different admixture groups was significantly in the opposite direction, when taking the uncertainty of the estimates into account, indicating that the presented results are not confounded by population structure (ESM Fig. 2b). Furthermore, we did not observe inflation of our test statistic (λ = 0.82, ESM Fig. 2c), confirming that the applied linear mixed model satisfactorily accounts for admixture and relatedness among the included individuals.

While our primary aim was to investigate the effect of the *TBC1D4*–physical activity interaction on 2 h plasma glucose, our second aim was to investigate the effect of the interaction on the probability of having type 2 diabetes. We included 240 individuals with type 2 diabetes (of these, 38 individuals were homozygous *TBC1D4* p.Arg684Ter variant carriers) and 1969 individuals with normal glucose tolerance as a control group (of these, 28 individuals were homozygous *TBC1D4* p.Arg684Ter variant carriers) in a logistic model. We found indication of an interaction effect, suggesting an attenuation in the association between the *TBC1D4* p.Arg684Ter risk variant and odds of type 2 diabetes per kJ kg^−1^ day^−1^ unit increase in daily physical activity (OR 0.99 [95% CI 0.97, 1.00], *p* = 0.11)]. To put this into context, the probability of having type 2 diabetes for a standard individual (40-year-old, male sex) who was a homozygous *TBC1D4* risk variant carrier and had an assumed physical activity energy expenditure of 50 kJ kg^−1^ day^−1^ (approximate median of the cohort) was 0.44. Notably, the probability for type 2 diabetes was reduced to 0.40 with an additional 1 h of moderate activity (60 kJ kg^−1^ day^−1^) and it was reduced to 0.35 with an additional 1 h of vigorous activity (77 kJ kg^−1^ day^−1^) per day (ESM Fig. 3a). We did not observe inflation of test statistic in the analysis based on the logistic model (λ = 1.11, ESM Fig. 3b).

Lastly, we investigated the effect of the *TBC1D4*–physical activity interaction on other cardiometabolic traits and found nominally significant effects on waist/hip ratio (β[interaction] = −0.00026 / [kJ kg^−1^ day^−1^], *p* = 0.047, *n* = 2872; ESM Table 2) and insulin sensitivity index (β[interaction] = 0.0024 / [kJ kg^−1^ day^−1^], *p* = 0.045, *n* = 2634; ESM Table 2).

## Discussion

In the present study of the Greenlandic population, we found that homozygous carriers of the common muscle-specific *TBC1D4* p.Arg684Ter loss-of-function variant benefit from physical activity, as shown by lower postprandial plasma glucose levels after an oral glucose load and reduced probability for developing type 2 diabetes. We also found that the effect of physical activity is markedly greater for these carriers.

In fact, the effect size of the gene–environment interaction between the common *TBC1D4* p.Arg684Ter loss-of-function variant and physical activity on 2 h plasma glucose levels in Greenlanders is remarkably large compared with previously identified gene–environment interactions for complex human traits [21–23]. Previous studies in individuals of European ancestry have examined interactions of type 2 diabetes-associated genetic variants and physical activity on metabolic disease traits with mixed results [24–26]. Here, we report that 1 h of moderate-paced walking or 1 h of high-intensity activity lowers 2 h plasma glucose levels by an additional ~0.2 mmol/l and up to ~0.7 mmol/l, respectively, compared with spending this hour at rest. To place our observed effect sizes of the *TBC1D4*–physical activity interaction on 2 h plasma glucose levels in Greenlandic individuals into context, we looked up the reported effect of lifestyle intervention on 2 h plasma glucose levels in previously conducted landmark studies. Specifically, the results of the Finish Diabetes Prevention Study showed that 1 year of intensive lifestyle intervention, including 30 min of moderate physical activity per day, diet changes and reduction of body weight, reduced 2 h plasma glucose levels by ~0.6 mmol/l more in the intervention group compared with the control group [11]. After 3 years of intensive lifestyle intervention, the difference in 2 h plasma glucose levels between the intervention group and the control group was reduced to ~0.4 mmol/l [11]. Remarkably, we derived a similar effect size for isolated habitual physical activity in an epidemiological setting. The large effect size of the *TBC1D4*–physical activity interaction on 2 h plasma glucose and the high frequency of the *TBC1D4* p.Arg684Ter loss-of-function variant in the indigenous Arctic population enables a direct translation of the benefit of physical activity to tailored reduction of postprandial glucose levels for a substantial part of the population (i.e. the homozygous variant carriers).

We found indication that physical activity may have a greater beneficial effect on some (i.e. type 2 diabetes, waist/hip ratio and insulin sensitivity index) but not all of the tested secondary outcomes in homozygous *TBC1D4* p.Arg684Ter loss-of-function carriers, compared with heterozygous carriers and non-carriers. However, the results relating to secondary outcomes need to be interpreted with caution since we present results without adjustment for multiple testing. Long-term follow-up is needed before being able to make conclusions on whether there is increased risk for CVD among *TBC1D4* risk variant carriers and whether physical activity may have beneficial effects on other cardiometabolic risk traits.

The discovery of gene–physical activity interactions on diabetes outcomes is challenging in commonly studied populations (such as European and Asian) and requires large sample sizes, acquired through large research biobanks or large-scale multicentre meta-analysis, that often exceed the sample size needed to identify main effects of genetic variants [21]. Therefore, our results add to the growing literature on genetic analysis in population isolates, such as the Greenlandic Inuit population. Such studies can provide a powerful approach to identify genetic variants, identify gene–environment interactions with clinical relevance, and gain knowledge into the molecular mechanisms of common disease traits [27].

Our finding of the physical activity-induced reduction of postprandial glucose levels in quasi muscle-specific human knockouts carrying the *TBC1D4* p.Arg684Ter variant may pave the way towards new insights into molecular mechanisms. The variant confers loss of expression of a long TBC1D4 isoform in skeletal muscle, while other insulin-sensitive tissues display normal expression of a short TBC1D4 isoform [4]. As a consequence, skeletal muscle expression levels of the long TBC1D4 isoform mRNA and protein are decreased with increasing number of *TBC1D4* p.Arg684Ter risk alleles, with homozygous carriers having practically no expression [4]. Our previous study of the human homozygous *TBC1D4* p.Arg684Ter variant carriers [4] and studies in *Tbc1d4*-knockout mice unequivocally suggest that lack of TBC1D4 lowers total GLUT4 protein content in specific muscle fibre types [28–31]. In addition, insulin-stimulated translocation of the GLUT4 transporter proteins to the plasma membrane might be compromised, albeit not to the same extent in different muscle fibre types [31]. The combination of lowered GLUT4 content and compromised insulin-stimulated GLUT4 translocation is suggested to lead to the postprandial hyperglycaemia and hyperinsulinaemia observed in the human homozygous *TBC1D4* p.Arg684Ter variant carriers [4, 5]. We speculate that the benefit of physical activity on lowering postprandial glucose levels in homozygous muscle-specific *TBC1D4* p.Arg684Ter loss-of-function carriers might be mediated via a TBC1D4-independent muscle-specific pathway and/or tissue crosstalk from muscle to other tissues that do have an intact TBC1D4 signalling pathway. One recent example of a study pointing towards inter-organ crosstalk showed that exercise training improves glucose tolerance through crosstalk between muscle and adipose tissue through an exercise-induced adipokine [32]. It may also be possible that a change in TBC1D1, which has also been implicated in contraction induced glucose uptake [33, 34], may contribute to the enhanced response to physical activity in homozygous carriers of the *TBC1D4* p.Arg684Ter loss-of-function variant.

We performed several sensitivity analyses that strengthen our findings. In the subset of individuals for whom we had information on objectively assessed physical activity we confirmed the direction and magnitude of the interaction effect. The lack of statistical significance in this sensitivity analysis is clearly a power issue, considering the loss, overall, of 1267 individuals (including 53 homozygous *TBC1D4* p.Arg684Ter variant carriers) compared with the analysis based on questionnaire-derived physical activity. Interestingly, the commonly used additive model showed that the *TBC1D4* p.Arg684Ter–physical activity interaction also had a significant effect on 2 h plasma glucose levels in the subset of individuals with objectively assessed physical activity. However, the magnitude of the interaction effect was overall smaller than the interaction effect derived from the primary model, likely due to heterozygous *TBC1D4* p.Arg684Ter variant carriers that are accounted for in an additive interaction model but not in the primary recessive model. Compared with homozygous carriers, in heterozygous carriers (more common in the Greenlandic population [4]) the *TBC1D4* p.Arg684Ter variant has less of an effect on metabolic traits, explaining the increased statistical power by applying an additive interaction model. Finally, the presented results are not confounded by population structure, the degree of admixture or relatedness among the Greenlandic population.

We were limited to the analysis of epidemiological data collected as part of a general health survey of the Greenlandic population and therefore the reported effect sizes need careful interpretation. To quantify the clinical impact of physical activity in carriers of the *TBC1D4* p.Arg684Ter loss-of-function variant and, based on this, recommend personalised treatment, an exercise-training intervention trial specifically targeted to Arctic individuals should be performed. Such a trial might include detailed OGTT measures of glucose homeostasis and type 2 diabetes remission as primary outcomes, as well as collection of tissue biopsies for functional and biochemical analysis. If designed properly, studies of human *TBC1D4* p.Arg684Ter loss-of-function variant carriers may also provide information on molecular mechanisms of the role of TBC1D4 in the beneficial effects of physical activity and determine whether TBC1D1 may play a contributary role in the enhanced response to physical activity in homozygous carriers of the *TBC1D4* p.Arg684Ter loss-of-function variant. We measured circulating glucose levels during an OGTT and interpreted our results as if skeletal muscle is the major tissue involved in exercise-induced priming of glucose uptake. However, we cannot exclude that other organs such as adipose tissue may play a role in the improved glucose tolerance in homozygous *TBC1D4* variant carriers who spend more time performing physical activity.

In summary, the results of our study suggest that regular moderate physical activity produces a clinically relevant improvement in postprandial glucose levels in indigenous Arctic individuals carrying two copies of the *TBC1D4* p.Arg684Ter loss-of-function variant. This provides a rationale to implement lifestyle intervention therapy by motivating a physically active lifestyle among homozygous carriers of this variant, constituting 3.8% of the population.

## Supplementary Information

ESM (PDF 889 kb)

## Data Availability

Relevant data for the present study are within the presented article and in the ESM. The Greenlandic Cardio-Metabochip data for the Inuit Health in Transition study has been deposited at the European Genome-phenome Archive (https://www.ebi.ac.uk/ega/dacs/EGAC00001000736) under accession EGAD00010001428. If you wish to see additional data, the authors confirm that, for approved reasons, some access restrictions apply. Data are available from the Novo Nordisk Foundation Center for Basic Metabolic Research; authors may be contacted at torben.hansen@sund.ku.dk.

## Abbreviations

MET: Metabolic equivalent task
RabGAP: Rab-GTPase-activating protein
TBC1D4: TBC1 domain family member 4
