# Supplementary material for: Physical activity attenuates postprandial hyperglycaemia in homozygous TBC1D4 loss-of-function mutation carriers
Source: Diabetologia. 2021 Apr 29;64(8):1795–804. doi: 10.1007/s00125-021-05461-z (PMC8245392; doi:10.1007/s00125-021-05461-z)
Supplement: Supplementary file 1 — (PDF 889 kb) [file 125_2021_5461_MOESM1_ESM.pdf]

Physical activity attenuates postprandial hyperglycemia in homozygous *TBC1D4* loss-of-function mutation carriers

Schnurr TM et al.

Electronic Supplementary Material (ESM)

ESM Methods 2

Cardiometabolic traits 2

International Physical Activity Questionnaire adapted to Greenlandic living conditions 2

Statistical analysis 2

Details on the applied linear mixed model 2

Details on the applied logistic model 3

ESM Tables 4

ESM Table 1. Clinical characteristics. 4

ESM Table 2. Interaction effect between the TBC1D4 p.Arg684Ter loss-of-function variant and physical activity on additional cardiometabolic traits in Greenlandic individuals. 6

ESM Figures 7

ESM Fig. 1. Flowchart overview of the inclusion N=2655 participants included in the primary main analysis. 7

ESM Fig. 2. Diagnostic plots of the interaction analysis between the *TBC1D4* p.Arg684Ter loss-of-function variant and physical activity on 2-hour plasma glucose levels. 8

ESM Fig. 3. Illustration of the *TBC1D4* p.Arg684Ter-physical activity interaction on the probability of being a type 2 diabetes case based on a standard individual (40-year old male). 10

ESM Methods

Cardiometabolic traits

Serum insulin levels were analysed using an immunoassay array, excluding des-31,32 split products and intact proinsulin (AutoDELFIA, PerkinElmer), while serum C-peptide levels were analysed using Cobas e411 (Roche). OGTT measures of plasma glucose and serum insulin were used to calculate insulin sensitivity index using following formula: Insulin sensitivity index = ([75,000 + (fasting glucose[mmolL^{-1}] × 18 – 2-hour glucose[mmolL^{-1}] × 18) × 0.19 × weight [kg]]/120)/[0.5(fasting glucose[mmolL^{-1}] × 18 + 2-hour glucose[mmolL^{-1}] × 18)]/1og[(fasting insulin[pmolL^{-1}]/6.945 + 2-hour insulin[pmolL^{-1}]/6.945)/2]. HbA1c was analysed by ion-exchange HPLC (G7, Tosoh Bioscience) or VARIANT (Bio-Rad). Fasting serum HDL-cholesterol, total cholesterol, and triglycerides were measured using enzymatic calorimetric techniques (Hitachi 917), while fasting LDL-cholesterol was calculated according to Friedewald's formula. Weight was measured on a standard electronic clinical scale and participants were wearing light indoor clothes. Waist circumference was measured midway between the rib cage and iliac crest, while hip circumference was measured at its maximum. Body fat percentage was calculated from bioimpedance measurements (Tanita, TBF-300MA). Blood pressure was measured three times and the two last measures were averaged for analysis.

International Physical Activity Questionnaire adapted to Greenlandic living conditions

Information about time spent on physical activity in the previous 7 days were collected using a face-to-face interview. As detailed elsewhere [1], the original English version of the long International Physical Activity Questionnaire (IPAQ) was translated into Greenlandic and backtranslated by two translators bilingual in Danish and Greenlandic and familiar with Greenlandic living conditions. During this assessment, the participants indicated how often (number of days per week) and for how long (average duration per day) they engaged in physical activity. This was done separately for vigorous intensity, moderate intensity, and walking in the domains work, transportation, domestic and leisure time. The questions were adjusted to arctic living conditions by culturally relevant examples. In the domestic domain, we combined the two questions concerning moderate intensity (outside and inside activity) into one. It was also considered that gardening is nonexistent in arctic living conditions, and common activities such as getting fishing equipment ready are done both inside and outside the house.

Statistical analysis

Details on the applied linear mixed model

We chose a fully recessive model for the interaction between *TBC1D4* p.Arg684Ter genotype and physical activity on 2-hour plasma glucose. We chose a model that allows for a 1-parameter test for interaction. Of the possible models that allow for a 1-parameter test for interaction, a recessive model seemed the most reasonable, since the main effect is close to being recessive. More specifically, we modelled the effects of genotypes, physical activity

and additional covariates, age and sex as fixed effects, and admixture and relatedness as random effects and derived the following analysis model:

$$y_i = \beta_0 + \beta_1 a_i + \beta_{HO}\, g_{HOi} + \beta_{HOxa}\, g_{HOi}\, a_i + \beta_{sex}\, sex_i + \beta_{age}\, age_i + u_i + e_i \qquad \textbf{[1]}$$

In this model, which we denote the primary analytical model, (**Equation [1]**), y_i is the phenotype for individual *i* (2-hour plasma glucose when nothing else is explicitly stated); β_0 is the intercept; a_i is physical activity in kJ kg^{-1} day^{-1} for individual *i* with an effect size of β_1 [beta (main effect of physical activity)]; g_{HOi} encoding whether individual *i* is homozygous carrier of the *TBC1D4* variant respectively with effect size β_{HO} [beta (main effect of being homozygous *TBC1D4* variant carrier)] respectively; β_{HOxa} [beta (interaction)] is the effect size of the interaction term for homozygous carriers; sex is coded 1 for males and 2 for females with effect size β_{sex}; age is the age of the individual with effect size β_{age}; e_i is the residual for individual *i,* the residuals are assumed to be independent and identically distributed $\sim N(0,\sigma)$; u_i is the *ith* element of the vector *u* of random effects modeling genetic similarity of individuals: $u \sim MVN(0, \lambda G)$; *G* is the genetic similarity matrix [1] and λ is a parameter used for scaling. Based on the primary analytical model we primarily tested the null hypothesis (H_0) that β_{HOxa} is 0, which corresponds to testing if the effect of activity on the phenotype differs significantly between homozygous carriers and the rest. The genetic similarity matrix was based on all 2655 individuals with available information on genotype, self-reported physical activity, 2-hour plasma glucose concentrations and covariates (the study population included in our main analysis). For this, we used SNPs with minor allele frequency > 1% and missingness < 5%. Furthermore, we considered a model very similar to the primary analytical model (**Equation [1]**) with the only difference being that BMI was included as additional covariate. Besides the recessive (primary) analytical model, we also considered a standard additive interaction model **[2]**, i.e. the model:

$$y_i = \beta_0 + \beta_a a_i + \beta_g\, g_i + \beta_{gxa}\, g_i\, a_i + \beta_{sex}\, sex_i + \beta_{age}\, age_i + u_i + e_i \qquad \textbf{[2]}$$

where g_i encodes how many copies of the *TBC1D4* variant individual *i* is carrying and; and where β_{gxa} is the effect size of the interaction term. For this model we tested the null hypotheses that β_{gxa} is 0.

Details on the applied logistic model

We also tested for an interaction between the *TBC1D4* variant and physical activity on type 2 diabetes using a logistic model implemented in GMMAT [2]. The applied model was similar to the primary analytical model derived in **Equation [1]**, where 2-hour plasma glucose levels were replaced by type 2 diabetes status.

ESM Tables

ESM Table 1. Clinical characteristics.

	N	Median (IQR)
N (men/women)*	2655 (1159/1496)	-
N (WT/HE/HO)*	2655 (1835/726/94)	-
Age (years)*	2655	44 (34-53)
**Body composition**		
BMI (kg/m^2)	2906	25.4 (22.4-29.0)
Waist (cm)	2875	88 (80-99)
Hip (cm)	2873	98 (92-104)
Waist-hip ratio	2872	0.90 (0.85-0.96)
Body fat percentage (%)	2588	27.9 (19.7-36.3)
**Blood pressure**		
Systolic	2517	122 (111-135)
Diastolic	2517	76 (69-84)
**Biochemistry**		
Fasting plasma glucose ($mmolL^{-1}$)	2742	5.6 (5.2-6.0)
2-hour plasma glucose ($mmolL^{-1}$)	2655	5.4 (4.3-6.7)
Fasting serum insulin ($pmolL^{-1}$)	2742	37 (25-56)
2-hour serum insulin ($pmolL^{-1}$)	2656	108 (47-210)
HbA1c ($mmolL^{-1}$)	2922	6.7 (6.2-7.1)
HbA1c (%)	2922	5.8 (5.5-6.1)
Fasting serum C-peptide ($pmolL^{-1}$)	2742	495 (357-691)
2-hour serum C-peptide ($pmolL^{-1}$)	2656	1680 (1086-2440)
Fasting serum LDL cholesterol ($mmolL^{-1}$)	2774	3.6 (2.9-4.3)
Fasting serum HDL cholesterol ($mmolL^{-1}$)	2933	1.6 (1.3-1.9)
Fasting serum total cholesterol ($mmolL^{-1}$)	2797	5.8 (5.1-6.6)
Fasting serum triglycerides ($mmolL^{-1}$)	2933	1.0 (0.8-1.4)
Insulin sensitivity index	2643	2.6 (1.9-4.0)
**Physical activity**		
Physical activity energy expenditure (International Physical Activity Questionnaire, kJ kg^{-1} day^{-1})	2933	47.3 (23.4-82.1)
Physical activity energy expenditure (combined heart rate and acceleration, kJ kg^{-1} day^{-1})	1388	48.7 (35.4-65.6)

Data are presented as medians followed by upper and lower quantile (inter quartile range, IQR) in parentheses. *Data presented for the N=2655 individuals with complete information

on *TBC1D4* genotype, 2-hour plasma glucose and questionnaire derived physical activity energy expenditure that were included in the main analysis.

Abbreviations: HO, Homozygous TBC1D4 p.Arg684Ter variant carriers; HE, heterozygous carriers; IQR, inter quartile range; WT, non-carriers

ESM Table 2. Interaction effect between the TBC1D4 p.Arg684Ter loss-of-function variant and physical activity on additional cardiometabolic traits in Greenlandic individuals.

Cardiometabolic trait	N	Effect (SD)	Std. Error	P-value	Effect [beta(interaction)] in (kJ kg^{-1} day^{-1})
**Body composition**					
BMI (kg/m^2)	**2906**	-0.0022	0.0020	0.28	-0.0090
Waist (cm)	**2875**	-0.0030	0.0020	0.13	-0.036
Hip (cm)	**2873**	-0.0017	0.0019	0.38	-0.010
Waist-hip ratio	**2872**	-0.0038	0.0019	0.047	-0.00026
Body fat percentage (%)	**2588**	-0.0019	0.0020	0.36	-0.017
**Blood pressure**					
Systolic	**2517**	0.0017	0.0020	0.40	0.031
Diastolic	**2517**	0.0025	0.0020	0.21	0.027
**Biochemistry**					
Fasting plasma glucose ($mmolL^{-1}$)	**2742**	0.00071	0.0019	0.71	0.0035
Fasting serum insulin ($pmolL^{-1}$)	**2742**	0.0019	0.0021	0.38	0.043
2-hour serum insulin ($pmolL^{-1}$)	**2656**	-0.0031	0.0021	0.14	-0.79
Fasting serum C-peptide ($pmolL^{-1}$)	**2742**	0.0016	0.0021	0.44	0.59
2-hour serum C-peptide ($pmolL^{-1}$)	**2656**	-0.0036	0.0019	0.061	-5.62
Insulin sensitivity index	**2634**	0.0041	0.0020	0.045	0.0024
HbA1c ($mmolL^{-1}$)	**2922**	3.3×10^{-5}	0.0017	0.98	0.0054
HbA1c (%)	**2922**	3.3×10^{-5}	0.0017	0.98	0.00049
Fasting serum LDL cholesterol ($mmolL^{-1}$)	**2774**	-0.00061	0.0019	0.76	-0.00035
Fasting serum HDL cholesterol ($mmolL^{-1}$)	**2933**	0.0014	0.0019	0.46	0.00076
Fasting serum total cholesterol ($mmolL^{-1}$)	**2797**	0.00055	0.0019	0.77	0.00082
Fasting serum triglycerides ($mmolL^{-1}$)	**2933**	0.0016	0.0020	0.43	0.0013

Effects (in SD of the study population), standard (Std.) errors and P-values are from analyses of of transformed traits. Effects [beta (interaction)] in (kJ kg^{-1} day^{-1}) are from analyses of untransformed traits. All results are presented from analyses of the primary analytical model (recessive model).

ESM Figures

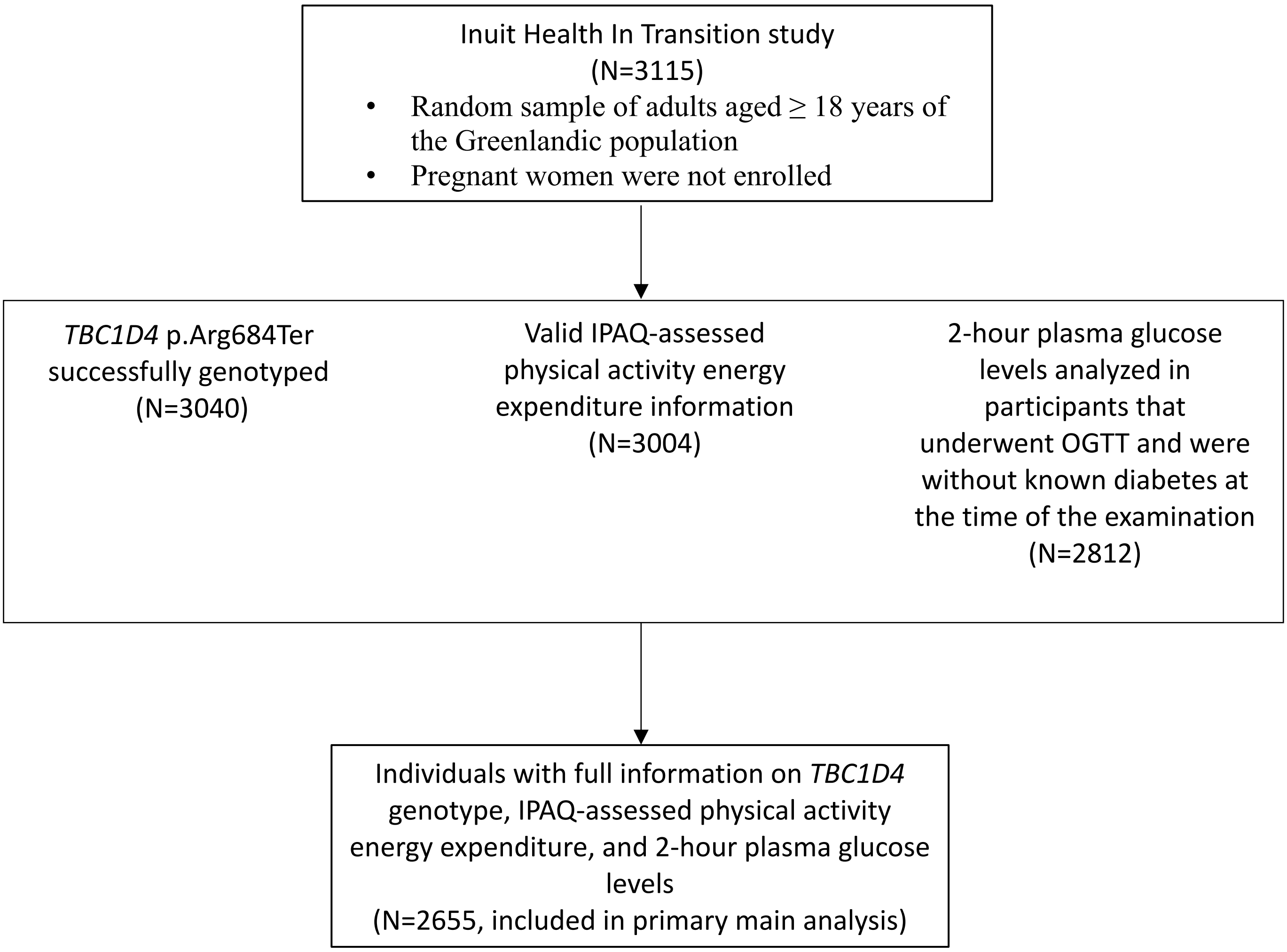

ESM Fig. 1. Flowchart overview of the inclusion N=2655 participants included in the primary main analysis.

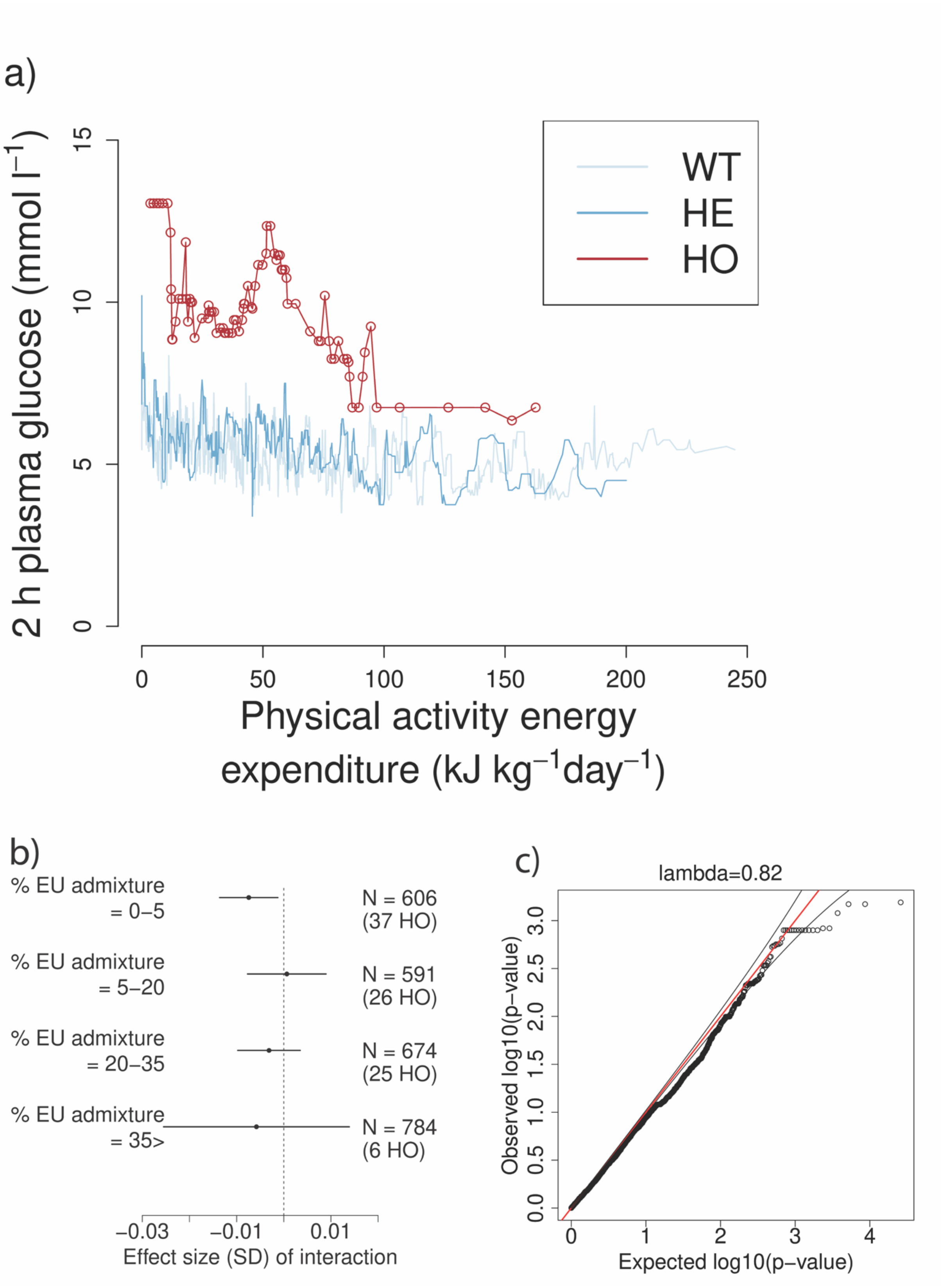

ESM Fig. 2. Diagnostic plots of the interaction analysis between the *TBC1D4* p.Arg684Ter loss-of-function variant and physical activity on 2-hour plasma glucose levels. a) Shows the decreasing trend for median 2-hour plasma glucose levels for the homozygous *TBC1D4* p.Arg684Ter risk variant carriers, while the 2-hour plasma glucose

levels for the non-carriers and heterozygotes remain relatively stable with increasing physical activity measured as physical activity energy expenditure (N=2655). Plots are based on raw values and do not take population structure into account. 2-hour plasma glucose levels and physical activity energy expenditure are displayed as median values derived by applying a sliding window algorithm with step size 10. **b)** Displays the effect of the interaction between the *TBC1D4* p.Arg684Ter loss-of-function variant carriers and physical activity on 2-hour plasma glucose levels, stratified by different degrees of admixture based on the primary model (**Equation [1], ESM Methods**). Effect sizes and 95% confidence intervals are shown for transformed data. Within each strata, the number of individuals (N) and homozygous risk carriers (HO) are indicated. Only the interaction effect size of the 0-5% EU admixture strata is significantly different from 0. This strata includes the largest proportion of homozygous risk carriers. For all other admixture categories, the interaction effect size does not deviate significantly in the opposite direction underlining that the degree of admixture does not confound the interaction analysis. **c)** Shows the QQ-plot of the observed –log10(p-values) [y-axis] versus the –log10(p-values) expected under the null hypothesis of no association [x-axis] from interaction analysis based on 18,807 randomly selected genetic variants on 2-hour plasma glucose levels based on a linear mixed model and by applying the additive model **Equation [2], ESM Methods**. The inflation factor lambda was 0.82 and is defined as the median test statistics divided by the expected median. This highlight that there is no systematic inflation of the applied test statistics.

Abbreviations: HO, Homozygous *TBC1D4* p.Arg684Ter variant carriers; HE, heterozygous carriers; WT non-carriers; % EU admixture, Estimated admixture proportions of European ancestry within Greenlanders; QQ, Quantile-quantile

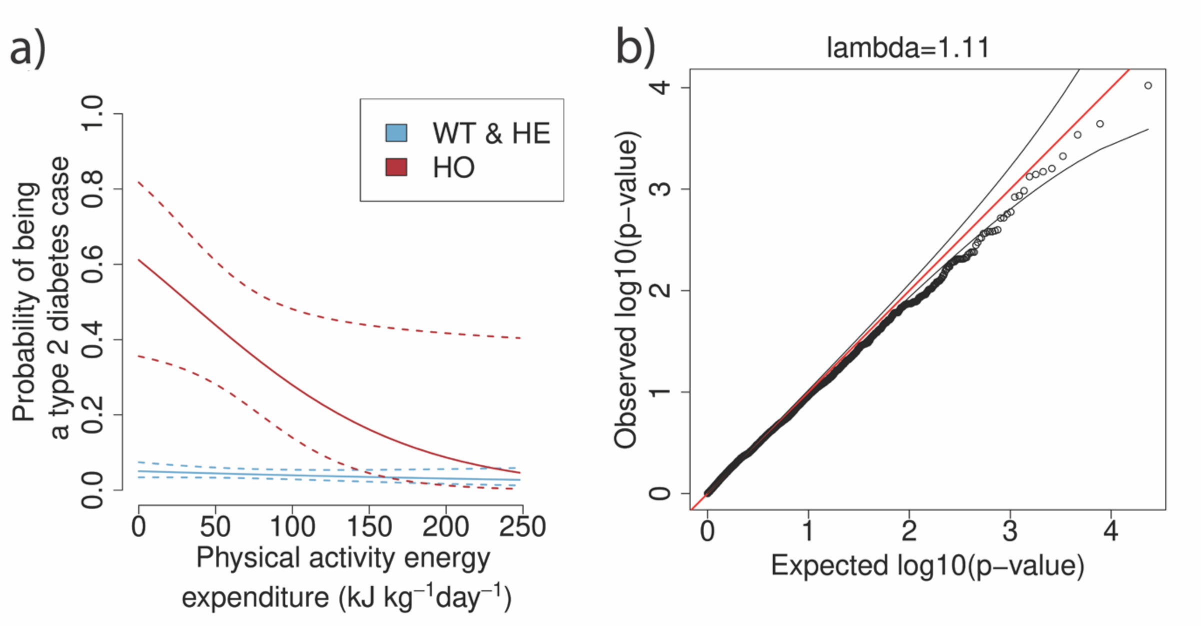

ESM Fig. 3. Illustration of the *TBC1D4* p.Arg684Ter-physical activity interaction on the probability of being a type 2 diabetes case based on a standard individual (40-year old male). a) Interaction effect between the *TBC1D4* p.Arg684Ter loss-of-function variant and physical activity measured as physical activity energy expenditure on type 2 diabetes status by genotype. For each individual, the respective physical activity energy expenditure is plotted on the x-axis. The y-axis displays the probability of being a type 2 diabetic case. The type 2 diabetes predictions were performed based on the given data of the Greenlandic cohort for a 40 year old male (defined as standard individual) and using the estimated effect sizes from the logistic regression model as described and derived in **ESM Methods**. **b)** The QQ-plot indicates that there was no systematic inflation of the applied test statistics for the logistic regression when testing for an interaction between the *TBC1D4* p.Arg684Ter loss-of-function variant and physical activity on type 2 diabetes status based on 5,607 randomly selected genetic variants (lambda = 1.1).

Abbreviations: HO, Homozygous *TBC1D4* p.Arg684Ter variant carriers; HE, heterozygous carriers; WT non-carriers; QQ, Quantile-quantile

ESM References

1. Dahl-Petersen IK, Hansen AW, Bjerregaard P, Jørgensen ME, Brage S (2013) Validity of the international physical activity questionnaire in the arctic. Med Sci Sports Exerc 45(4):728–736
2. Chen H, Wang C, Conomos MP, et al (2016) Control for Population Structure and Relatedness for Binary Traits in Genetic Association Studies via Logistic Mixed Models. Am J Hum Genet 98(4):653–66. https://doi.org/10.1016/j.ajhg.2016.02.012
